# 

**Published:** 1860-07

**Authors:** 


NORTH AMERICAN

### MEDICO-CHIRURGICAL REVIEW.

Vol. IV.] JULY, 1860. [No. 4.

### CONTENTS.

### ANALYTICAL AND CRITICAL REVIEWS.

| Art. | | Page |
|---|---|---|
| I. | —A Practical Treatise on Fractures and Dislocations. By Frank Hastings Hamilton, M.D. | 577 |
| II. | —Therapeutics and Materia Medica. By Alfred Stillé, M.D. | 588 |
| III. | —Footfalls on the Boundary of Another World. By Robert Dale Owen | 615 |
| IV. | —On the Injurious Effects of Mercury in the Treatment of Disease. By S. O. Habershon, M.D. | 632 |
| V. | —Transactions of the American Medical Association | 639 |
| VI. | —Lectures on Pathological Anatomy. By Samuel Wilks, M.D. | 642 |
| VII. | —Nature in Disease ; illustrated by various Discourses and Essays. By Jacob Bigelow, M.D. | 643 |
| VIII. | —Hæmorrhoids and Prolapsus of the Rectum; their Pathology and Treatment. By Henry Smith, F.R.C.S. | 644 |
| IX. | —The New Sydenham Society's Publications for 1859 | 645 |

### ORIGINAL COMMUNICATIONS.

| | | |
|---|---|---|
| I. | —Notes of Selected Cases from the Wards of the Charity Hospital, at New Orleans. By T. G. Richardson, M.D. | 646 |
| II. | —Statistics of Insanity in the United States. By Richard J. Dunglison, M.D. | 656 |
| III. | —Practical Observations upon the Nature and Treatment of Prostatorrhœa. By S. D. Gross, M.D. | 693 |

### BI-MONTHLY ABSTRACTS.

SURGERY. BY S. W. GROSS, M.D.

On Lithotomy, considered as a Cause of Death; with Remarks on the Rectangular Operation.—New Operation for the Removal of Naso-pharyngeal Polyps.—Ligature of the Femoral Artery for the Cure of Elephantiasis.—On the Means to be employed in Syncope from Hemorrhage after Surgical Operations.—Secondary Amputations after Gunshot Wounds.—Treatment of Certain Diseased Conditions of the Hip-Joint.—Severe Burn followed by Exfoliation of the upper portion of the Skull.—Treatment of Cystic Goitre.—Cure of a Gastric Fistule by a Plastic Operation. 700

PATHOLOGY AND PRACTICE OF MEDICINE. BY RICHARD J. DUNGLISON, M.D.

Diagnosis and Treatment of Special Forms of Paralysis.—On the Atheromatous Expression.—Importance of the Functions of the Skin in Tubercular Consumption.—Contraction and Obliteration of the Aorta near the Junction of the Ductus Arteriosus.—Unsuccessful and Successful Attempts to produce Variola in the Cow.—On a form of Hypochondriacal Delirium. 708

OBSTETRICS, AND DISEASES OF WOMEN AND CHILDREN. BY WM. B. ATKINSON, M.D.

PAGE

Alum in the Treatment of Uterine Hemorrhage.—Morning Sickness: its Significance as a Symptom.—Occlusion of the Os Uteri during Labor.—Gangrene of the Uterus and Placenta.—Rupture of the Uterus, and Ventral Hernia during Pregnancy.—Displacements of the Uterus.—Puerperal Convulsions successfully treated by Injections of Morphia.—Collyrium in the Ophthalmia of New-born Infants ........ 717

PHYSIOLOGY. BY S. WEIR MITCHELL, M.D.

Observations on Digestion.—Experimental Epicrisis of some late Researches on Liver Sugar.—The Coccygeal Gland of Man ........ 724

MATERIA MEDICA AND THERAPEUTICS; MEDICAL CHEMISTRY; TOXICOLOGY AND LEGAL MEDICINE. BY EMIL FISCHER, M.D.

*Materia Medica and Therapeutics:* On Penghawar Djambi.—Employment of Santonine in Nervous Affections of the Eye.—Subcutaneous Injections of Sulphate of Atropine in Tetanus.—Glycerin in the Preparation of Sinapisms.—Pepsine in the Obstinate Vomiting of Pregnant Women.—Iodized Oil of Juniper-Berries.—Indian Hemp.—Cutaneous Eruptions following the Use of Iodine.—Gelsemin.—Formula for Ozanam's Solution of Bromine.—Sulphate of Quinia in Acute Articular Rheumatism.—Tannin as Antidote to Strychnine ........ 736

*Medical Chemistry:* Mode of Estimating the Quantity of Albumen in Albuminous Fluids.—On the Composition of the Cerebral Substance ........ 746

*Toxicology and Legal Medicine:* Poisoning by Arsenic.—Detection of Metallic Poisons by means of Electrolysis.—Poisoning by Strychnia ........ 747

PATHOLOGICAL ANATOMY. BY JOHN H. PACKARD, M.D.

Pathology of the Pituitary Body.—Medullary Fungus of the Pineal Gland.—Contraction and Obliteration of the Aorta near the Junction of the Ductus Arteriosus.—Hydatid Tumor in the Apex of the Right Ventricle of the Heart.—Abscess in the Heart.—Hydatid Tumor and Abscess of the Liver.—Rupture of the Spleen from a Trifling Cause.—General Phlegmon of the Abdomen.—Cases of Renal Abnormity ........ 750

EDITORS' TABLE.—The Laryngoscope.—Japanese Physicians.—Middle Georgia Medical College.—National Convention for Revising the Pharmacopœia of the United States.—Children's Hospital of Philadelphia.—Kentucky Asylum for Feeble-minded Children.—Pennsylvania Training School for Feeble-minded Children.—Army Medical School.—University of Pennsylvania.—Naval Medical Appointments.—Medical Society of the State of Pennsylvania.—Medical Journals.—Dr. E. Brown-Séquard.—Memorial to John Hunter.—Physiology.—Dinner to Dr. George B. Wood.—American Medical Association.—Convention of Superintendents of Hospitals for the Insane.—New Medical College.—To Correspondents ........ 759

NECROLOGICAL NOTICES.—Mr. James Braid.—Dr. John Lizars.—Professor Guislau.—Professor Andreas Retzius ........ 767

BI-MONTHLY BIBLIOGRAPHICAL RECORD ........ 768

